# Quantifying Oldowan Stone Tool Production at Olduvai Gorge, Tanzania

**DOI:** 10.1371/journal.pone.0147352

**Published:** 2016-01-25

**Authors:** Jay S. Reti

**Affiliations:** Department of Anthropology, University of California Santa Cruz, Santa Cruz, California, United States of America; University of Oxford, UNITED KINGDOM

## Abstract

Recent research suggests that variation exists among and between Oldowan stone tool assemblages. Oldowan variation might represent differential constraints on raw materials used to produce these stone implements. Alternatively, variation among Oldowan assemblages could represent different methods that Oldowan producing hominins utilized to produce these lithic implements. Identifying differential patterns of stone tool production within the Oldowan has implications for assessing how stone tool technology evolved, how traditions of lithic production might have been culturally transmitted, and for defining the timing and scope of these evolutionary events. At present there is no null model to predict what morphological variation in the Oldowan should look like. Without such a model, quantifying whether Oldowan assemblages vary due to raw material constraints or whether they vary due to differences in production technique is not possible. This research establishes a null model for Oldowan lithic artifact morphological variation. To establish these expectations this research 1) models the expected range of variation through large scale reduction experiments, 2) develops an algorithm to categorize archaeological flakes based on how they are produced, and 3) statistically assesses the methods of production behavior used by Oldowan producing hominins at the site of DK from Olduvai Gorge, Tanzania via the experimental model. Results indicate that a subset of quartzite flakes deviate from the null expectations in a manner that demonstrates efficiency in flake manufacture, while some basalt flakes deviate from null expectations in a manner that demonstrates inefficiency in flake manufacture. The simultaneous presence of efficiency in stone tool production for one raw material (quartzite) and inefficiency in stone tool production for another raw material (basalt) suggests that Oldowan producing hominins at DK were able to mediate the economic costs associated with stone tool procurement by utilizing high-cost materials more efficiently than is expected and low-cost materials in an inefficient manner.

## Introduction

The Oldowan stone tool industry has traditionally been described as a simple technology [1,2] with variation stemming from raw material constraints [3–5]. For the period of human evolution between 2.5 and 1.5 million years ago, Oldowan lithic artifacts remain a primary indicator of human behavior. Though technological homogeneity within the Oldowan has been questioned [6–8], Oldowan research has primarily investigated research questions only tangentially related to its production. Productive research programs asking questions related to differential landscape usage [9–14] have demonstrated that Oldowan-producing hominins were cognizant of raw material economics. Similarly, even from the earliest Oldowan examples at Gona, Ethiopia [15,16], and Kanjera, Kenya [17] it is clear that early stone tool using hominins had preferences for some raw materials over others. Particularly successful research has focused on the functional aspects of Oldowan technology as related to cut-marked bone in both archaeological and experimental settings [10,18–27].

Several research programs address patterns in Oldowan stone tool morphology. Toth [3,4,28] transitioned the perception of Oldowan technology from a core-tool technology to a primarily flake-tool technology. His experimental work demonstrated the potential utility of using platform and dorsal cortex as markers of reduction intensity. More recently, Roche and colleagues argued that Oldowan technology is not as uniform as was once thought based on refitted artifacts from Lokalalei 2C from the west side of Lake Turkana in Northern Kenya [29,30]. Clear evidence suggests that later Oldowan assemblages at Peninj have curated components [31] and that Olduvai Gorge assemblages were utilized for diverse functions [32], though other research suggests more uniformity in these functions [10]. However, early lithic assemblages from Gona, Ethiopia may suggest technological stasis in the Oldowan [33]. Linear regression models of Oldowan reduction demonstrate efficacy in statistically predicting the placement of individual Oldowan flakes within an Oldowan reduction sequence [13]. Such statistical techniques allow for more accurate assessments of reduction intensity within the Oldowan.

Despite recent progress toward re-evaluating Oldowan technology, research has not addressed the possibility of statistically quantifying production behaviors, that is, the differential methods by which flakes were created. If variation exists within Oldowan technology, as many suggest, where does it exist? Is it in how Oldowan sites were used, how raw materials were utilized, how the stone tools were actually manufactured [7,8], or a combination of these factors? Further, can variation be attributed to differences in how raw materials fracture [34,35] or can variation be attributed to behavioral patterns in hominin production, skill, and economics?

While refitted flakes and cores from Lokalalei is informative for that site [29], few other sites currently exist that have such refits. Further, lithic artifacts from Lokalalei were produced on different raw materials than sites from Koobi Fora on the East side of Lake Turkana, let alone other Oldowan localities, and it has not been demonstrated how, or if, hominins differentially interacted with the raw material utilized for stone tool production. Thus the comparative value of Lokalalei to other Oldowan sites is, at least at present, limited. Experimental methods, however, allow for a more definitive model of Oldowan behavior since the methods used to produce the stone tools are empirically known [13]. Such experimental models require large sample sizes of flakes to establish expectations for archaeological variation. Large samples of cores are necessary to determine the impact that differing core morphologies have on resulting flakes throughout the process of reduction. Such a large scale, quantitative experimental model have yet to be undertaken, but the potential to statistically identify, assess, and compare the production behaviors associated with Oldowan stone tool manufacture is, nonetheless, there.

The research presented here establishes a large, controlled experimental assemblage that establishes four quantifiable production behaviors for Oldowan stone tools. Such a methodology adds a standardized research strategy to Oldowan studies that statistically identifies how individual stone tools were manufactured and allows for different Oldowan assemblages to be compared in a behaviorally informative manner; by comparing flakes made via specific production behaviors, the statistical comparison is between manufacturing strategies, not raw materials. Thus raw material constraints are no longer a burden for comparison. Further, this method allows research to focus on questions such as whether or not Oldowan producing hominins were making stone tools in the same way across paleolandscapes. If so, how was this production technique maintained across these landscapes? If not, can technological divergence within the Oldowan help to explain technological evolution on a broader level? Answering questions such as these is possible and is necessary to explain both the origins of lithic technology and the evolution of technology through time [32,33,36]. Adding this dialogue to Oldowan research contributes to the broader discussion of early human behavior as determined from faunal analytical methods, geological methods, and raw material procurement and functional studies.

## Materials and Methods

To establish a null model for Oldowan morphological variation, experimental archaeological replication is used. Experimental stone tool production has a long history as an archaeological tool [3,4,7,8,28,34,35,37–48]. The term “flintknapping” refers to the production of stone tools generally, such that modern day enthusiasts are called “flintknappers.” This contrasts with the scientific concept of “replication,” which refers to controlled methods that seek to accurately model archaeological phenomena. Research related to replication analyses seeks to produce flakes with as much control as possible to model production behaviors utilized by stone tool producing populations. This is not to say that replication studies are exact replicas, or that analysts assume them to be directly analogous to archaeological specimens; rather, replicated materials represent assemblages with known sets of controlled conditions surrounding their manufacture. Replicated assemblages, therefore, allow for the experimental creation of a null model for archaeological material given a certain set of conditions. Given the conditions used during replication experiments, archaeological materials are statistically assessed to determine whether or not they fit within expectations or fall outside of expectations.

The null model that is developed here uses “least effort” assumptions as its controlled experimental conditions. Viewing the Oldowan as a simplistic technology has led to body of literature that models stone tool manufacture during this time period through a “least effort approach” to manufacture [3,10,13,29,48–52]. Isaac [52] suggested that the least effort approach be used as a null model before higher levels of inferences were applied to Oldowan assemblages, though no null model has yet to be effectively produced [53]. The least effort model assumes that stone tools were being produced with single flake production in mind; forethought to core shape and form is not considered under the least effort approach. Experimentally, the sole concern of replication experiments is to produce flakes. Using this approach there is no distinct “reduction sequence” for any given core. Rather, flakes are removed as independent behavioral events, each determined by the core morphology at the time of its creation. Each flake removal, however, has a particular production behavior, defined in subsequent sections, associated with its manufacture.

### Raw material selection

To model Oldowan production methods at Olduvai Gorge, raw materials utilized by Oldowan producing hominins at Olduvai Gorge were used as experimental samples. In this way replication experiments model the actual fracture mechanics that the population in question faced when producing stone tools.

During the Oldowan of Olduvai Gorge, two dominant materials were used to produce lithic implements: volcanic basalt and quartzite. While a large basaltic layer underlies Bed I at Olduvai Gorge [54], hominins were not likely utilizing these basement basalts for stone tool manufacture. Instead, hominins collected basalts in the local streams and channels that originated from the volcanic highlands to the east. The cobbles of basalt fluvially transported from these volcanic highlands to the areas surrounding the paleolake at Olduvai were naturally worn, smaller in size, and generally more acceptable for stone tool production. Cobbles in the modern Olduvai riverbeds were assessed and collected for experimental use under the permission of the Tanzania Commission for Science and technology (COSTECH) and exported under the authority of the Tanzania Revenue Authority, Customs and Excise.

**Fig 1:**
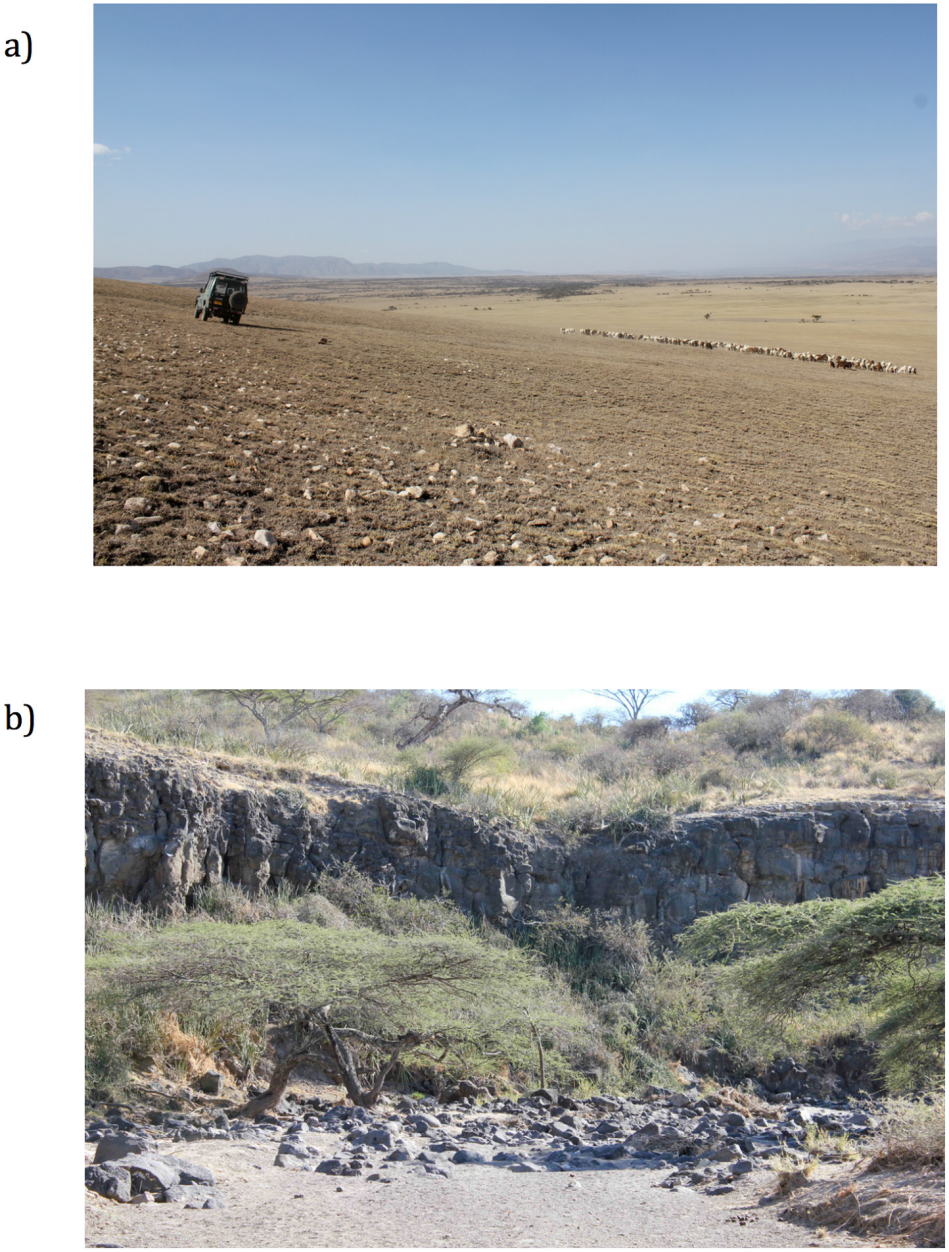
Olduvai Gorge, Tanzania raw material sources. a.) Naibor Soit is the source of quartzite from Olduvai Gorge. b) Modern exposure of basement basalts occur at locality KK and cobbles of basalt are available for procurement in the adjacent river bed. Basalt cobbles such as these are eroded from the volcanic highlands to the East of Olduvai Gorge.

Quartzite outcrops at the large inselberg called Naibor Soit (Fig 1). Naibor Soit is the closest source to Oldowan archaeological sites and is the most likely raw material source for the quartzite utilized at these locations [20,55]. Eroded cobbles of this thick, tabular quartzite were assessed for suitability and collected. Suitable quartzite cobbles ranged in size from several hundred grams to approximately two kilograms, had acceptable angular platforms available, and were of suitable quality to the naked eye.

Hammerstone selection was empirically determined through archaeological assemblage composition and consisted of both basalt and quartizite hammers. Based on the material and weight of excavated hammerstones, cobbles of equivalent weight and raw material were selected for experimental use. Multiple hammerstones were utilized during reduction experiments and were naturally rounded cobbles collected from the Olduvai river channel. Experimental hammerstones varied around the mean weight of the archaeological hammerstone sample and included hammers near the mean weight, near one standard deviation above the mean, and near one standard deviation below the mean of the archaeological sample. For research conducted in this study, hammers near two standard deviations above the mean were also utilized to demonstrate whether hammer weight statistically affects resulting flake morphologies for these raw materials.

### Sample size

Many Oldowan replication experiments have been conducted, but with few exceptions [3] the experimental sample size is quite small [13,56]. While small experimental sample sizes can yield significant and important results [13], these samples are problematic when the results are applied archaeologically for several reasons. First, even when using a single raw material, the internal quality of that raw material can differ significantly from one cobble to another and this will affect how those cobbles fracture. Second, variation in initial cobble size might affect the morphologies of flakes that result from reduction of that cobble. Third, variation in the initial cobble shape can dramatically affect how flakes are removed from that core and in the morphology of the resulting flakes. Fourth, variation in hammerstone size and material may play a role in determining the resulting flake morphologies. Each of these aspects of experimental reduction must be statistically addressed in order to determine the efficacy of experimental methods to model archaeological behaviors.

Reduction experiments are, therefore, tedious because they should include a large sample size of reduced cores in order to produce an assemblage large enough to statistically assess the natural variation produced from core shape, core size, variation in raw material quality, and hammerstone variation. Previous replicative research has limited experiments to approximately 30 cobbles. The research presented here includes the systematic reduction of 219 cobbles to yield 1758 total detached pieces. This sample size of both cores and flakes is statistically large enough to assess whether differences in core morphology and material are potential reasons for morphological variation in flakes or whether this variation might be attributed to differences in production behaviors among hominins.

### Identifying production behaviors in the Oldowan

Quantifying how the Oldowan was manufactured necessitates being able to identify specific behaviors that Oldowan producing hominins employed when creating lithic implements. Statistically identifying such production behaviors in the Oldowan faces some potential analytical problems. Oldowan production patterns have traditionally been considered simple and expedient in nature [2,10,50,51,57], so a large range of variation in flake morphology is expected. Statistically identifying evidence of differential production behaviors may be difficult due to this broad range of variability. Any method that identifies such patterns should also have a margin of error associated with such classifications to provide an assessment of certainty in those classifications.

The methods outlined here use production behaviors to define how stone tools were manufactured. “Production” refers to the actual manufacture of the flake in question; “behavior” refers to the combination of 1) the cognitive understanding necessary to produce flakes in a particular way and 2) the ability to successfully remove the intended flake. Four production behaviors were experimentally identified that explain the behavioral suite behind how all Oldowan core forms were manufactured via a least effort approach.

#### Oldowan Behavior A (OBA)

**Fig 2:**
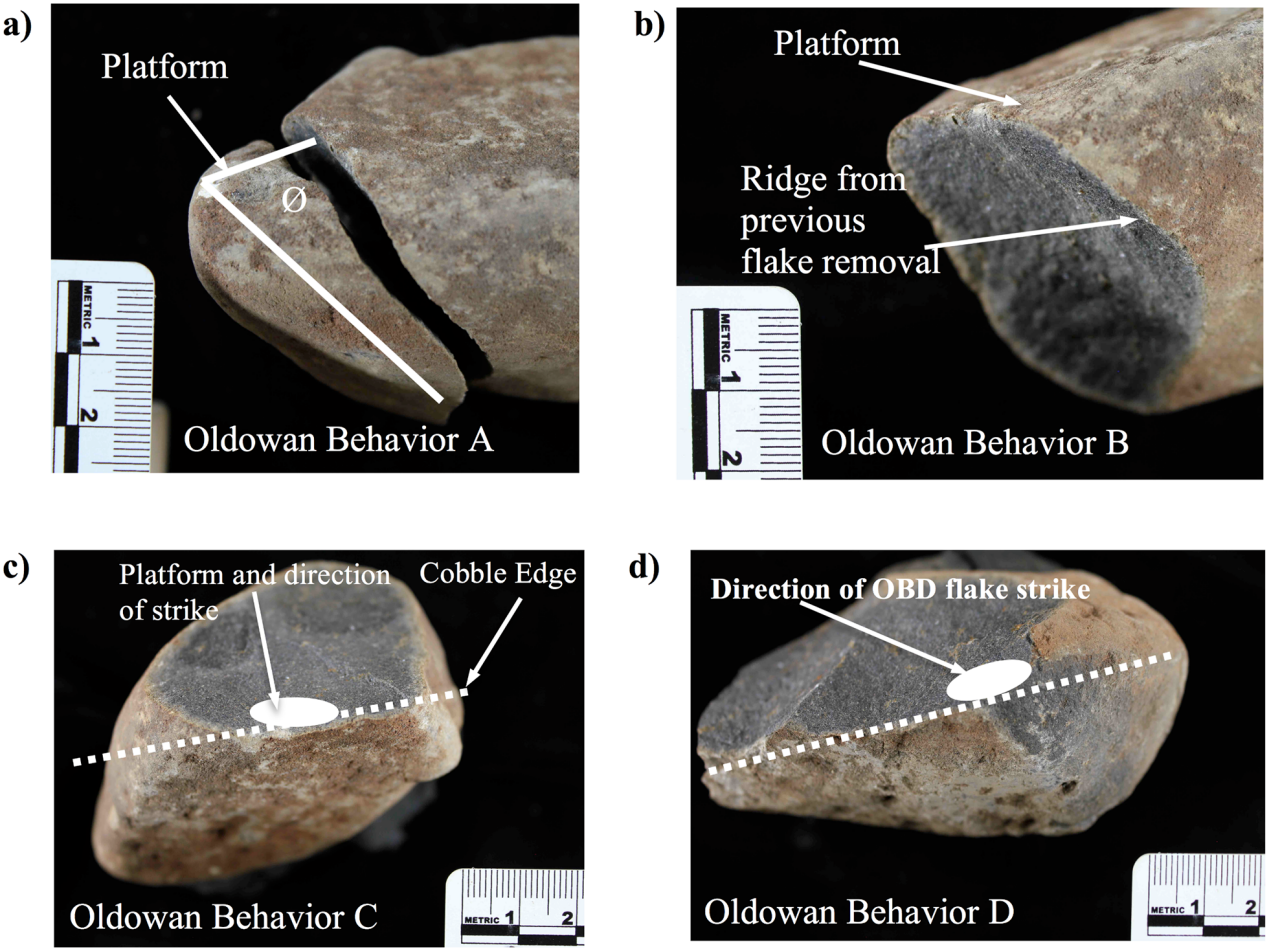
Oldowan production behaviors A-D. a) The primary prerequisite knowledge for flake production is the identification of an appropriate platform. Oldowan Behavior A (OBA) is the identification of an acutely angled edge on the cobble. b) Oldowan Behavior B (OBB) requires placement of a platform above a scar made from a previously removed flake (such as an OBA flake, though the process assumes no particular order in behaviors). c) Oldowan Behavior C (OBC) is bifacial flaking, using the noncortical surface produced from previous flake scars as the platform for the flake. d) Oldowan Behavior D (OBD) requires an obliquely struck flake relative to the cobble’s edge (represented by the dotted white line). Striking in such a direction removes a flake along the perimeter of the core instead of removing a flake toward the center of the core (as in OBC flakes).

To intentionally produce a flake via hard-hammer percussion, one primary piece of knowledge concerning fracture mechanics is cognitively necessary: an acute angle at the edge of the rock is needed for successful flake production. Conchoidal fracture requires that the force that is put into a particular rock via percussive action exits that rock. If a cobble is struck along an obtusely angled edge, or in the center of that piece of material, it is much more difficult to produce a predictable flake, if at all. Oldowan Behavior A (OBA) is a flake that has been produced by striking anywhere on an unmodified surface that forms an acute angle along the edge of that cobble (Fig 2a). To consistently remove flakes, as Oldowan producing hominins did, this fundamental principle was understood and successfully implemented.

#### Oldowan Behavior B (OBB)

Using a least effort model for flake production, once a flake has been produced using OBA the stone tool producer reevaluates the core and finds a desirable, acutely-angled platform to produce the next flake. This platform might be an unmodified, acutely angled portion of the core that is unrelated to the initial flake removal, in which case the resulting behavior would still be OBA. Predictability of flake production, however, increases when a platform is placed above the ridge produced from a previous flake removal. Cognitively understanding this aspect of fracture mechanics results in Oldowan Behavior B (OBB). The platform utilized for OBB is adjacent to a previously utilized cortical platform that produced another flake. The ridges on either side of this flake scar produce areas of relatively higher mass and also provide directionality for the force of the hammerstone strike to follow (Fig 2b). Removal of several flakes in this manner produces a “unifacial chopper,” using the Leakey typology.

#### Oldowan Behavior C (OBC)

Following either an OBA or OBB flaking event (or any combination thereof), the stone tool producer may begin to bifacially flake the core. Bifacial flaking requires that the core be flipped over such that a flake scar produced from previous flake removals becomes the platform for the next flake. Oldowan Behavior C (OBC) requires an understanding by the stone tool producer that flake scars are acceptable platforms for further flake removal and also requires a complex rotation of the core. Removal of OBA, OBB, and OBC flakes from the same edge of a core produces a core form that the Leakey typology refers to as a “bifacial chopper.”

#### Oldowan Behavior D (OBD)

The combination of OBA, OBB, and OBC flakes produces a bifacially flaked core. This kind of bifacial flaking removes flakes directly toward the midline of the core. Flaking limited to this kind of bifacial flake removal will result in a relatively blocky and thick core that becomes difficult to flake. Instead of focusing the direction of the hammerstone strike directly toward the midline of the core, as with OBB and OBC flaking events, Oldowan producing hominins were capable of directing the force of their hammerstone blows at an angle that propagated flakes around the perimeter of the core. This behavior of directing flake removals around the core’s perimeter designates Oldowan Behavior D (OBD). Several flakes removed in succession produces a characteristic “zig-zag” edge along the core that is particularly diagnostic of discoids (using the Leakey typology).

If a platform is available on a surface that produces a flake going in a direction other than the two bifacial directions already established on a core, the resulting core form is referred to as a “polyhedron” under the Leakey typology. However, no new behavior is necessary to produce this core form. Rather, if a cobble is thick and/or blocky to begin with, an OBB or an OBC behavior, directed in a new direction, is responsible for polyhedron production.

Each of these behaviors builds upon the cognitive understanding of the other behaviors and demonstrates a clear comprehension of fracture mechanics and an ability on the part of the Oldowan hominin responsible to actualize their intention. However, using a least effort approach to flake production no single behavior necessitates that another behavior follows; behaviors are utilized based only on what platforms are available. Further, a least effort approach necessarily means that no single “reduction sequence” can be applied to the Oldowan. Each core will yield a different set of behaviors and could change depending on the success or failure of each flake removal attempt.

### Replication experiments

Replication experiments were conducted over a canvas floor cover so that every fragment of debris was accounted for during stone tool production. Stone tool production was conducted such that the stone tool producer (J. S. Reti) was in an upright, seated posture. Experiments were conducted using freehand, hard hammer percussion; the core was held in the left hand and the hammer in the right hand. No gloves were used in the production of these experimental assemblages. The results presented in this paper are derived from the production behaviors associated with each flake.

Each cobble was measured in three dimensions prior to experimental reduction (Table 1). Each cobble was also weighed to the nearest 0.1 gram. The core was then photographed on three sides. The combination of these measurements and photographs permanently records the initial morphology of the core prior to reduction and thus provides a measure of whether or not initial core morphology affects resulting flake morphologies (see Fig E and Tables C-E in S1 File). Results show no statistical relationship between initial core morphology and the number of flakes resulting from that initial core morphology for either Olduvai Gorge quartzite or Olduvai Gorge basalt (Kruskal-Wallace test for multiple comparisons: quartzite p = 0.24, basalt p = 0.26, see Table E in S1 File for Tukey’s post-hoc test results, which also show no significance).

**Table 1:** Measurements taken on whole flakes and corresponding definitions.

Measurement	Definition
Length	Technological axis measured perpendicularly to the point of percussion, taken on the ventral side
Width	Perpendicular to length, measured at ½ length measurement, taken on the ventral side
Thickness	Measured in the third dimension at the point where width and length intersect
Bulbar Thickness	Measured in the same dimension as thickness but from the point of percussion
Platform Width	Measured from left lateral margin of the platform to the right lateral margin of the platform; measured along the same axis as width
Left Platform Width	Measured on the same axis as flake width but from the point of percussion to the furthest left margin of the platform
Right Platform Width	Measured on the same axis as flake width but from the point of percussion to the furthest right margin of the platform
Platform Thickness	Measured perpendicular to platform width and taken from the point of percussion to the furthest dorsal margin of the platform
Left Platform Thickness	Platform thickness measured at 1/5 the platform width
Right Platform Thickness	Platform thickness measured at 4/5 the platform width
Maximum Dimension	The longest dimension of the flake, taken on the ventral side
Platform Cortex	The percentage of the striking platform surface with cortex present
Dorsal Cortex	The percentage of the dorsal surface with cortex present
Platform Area	See Figs A-D and Tables A and B in S1 File.
Core length	The longest axis of the cobble
Core width	The longest axis perpendicular to the axis determined for maximum length
Core thickness	The maximum dimension that is perpendicular to both the length and the width axes

Each core was assessed to determine potential acceptable locations for the first platform. Acceptable platform location was determined for this primary flake solely by the availability of a proper angle at the edge of the core, as described in the methodology surrounding identification of OBA. The production behavior (OBA, OBB, OBC, or OBD) associated with each flake removal was recorded for each cataloged flake, linking each flake with a particular behavior and thus, a particular morphology with a particular production event. Flakes were removed from each core, utilizing the least effort approach to flake manufacture until the core was determined to be “exhausted.” An exhausted core was determined via one of two criteria: 1) no acceptable platforms remained for flake production or 2) based on hammerstone size, no platforms were available in which the stone tool producer would be successful in flake removal. If a core was determined to have significant impurities or fracture planes that prevented it from fracturing conchoidally, that cobble was removed from the sample.

### Measurements and Size Standardization

Each replicated flake was measured following production in the dimensions defined in Table 1. Only whole flakes were measured because each of the variables measured is complete in whole flakes. Any flake with missing measurements was excluded from analysis. Split, snapped, and split and snapped flakes were included only if all pieces were present and could be accurately measured.

Identical production behaviors can potentially produce flakes of very different sizes. For instance, when flaking a large cobble the first step to identifying an appropriate platform is to find an acute angle near the edge of that cobble. Using only this criterion to produce a flake, a flintknapper could remove a relatively large flake from a large cobble. Using the same criterion to produce a flake from a relatively small cobble, the same flintknapper might produce a relatively small flake. The behavior related to the production of both flakes is identical, yet the flakes are morphologically distinct based on size. All measurements of flakes were, therefore, size standardized to remove size-dependent bias from statistical analyses. Traditional size standardization techniques in lithic analysis have relied on using measurements assumed to approximate size to standardize all variables. However, using measures such as length, maximum dimension, or weight, to size standardize has been criticized in evolutionary biology due to constraints of the sample size, deviations from normal distributions within assemblages, consistency in fitting lines of regression, and allometric issues [58–60]. To avoid such problems, flakes were size standardized using their geometric mean [61].

### Classification algorithm construction

**Fig 3:**
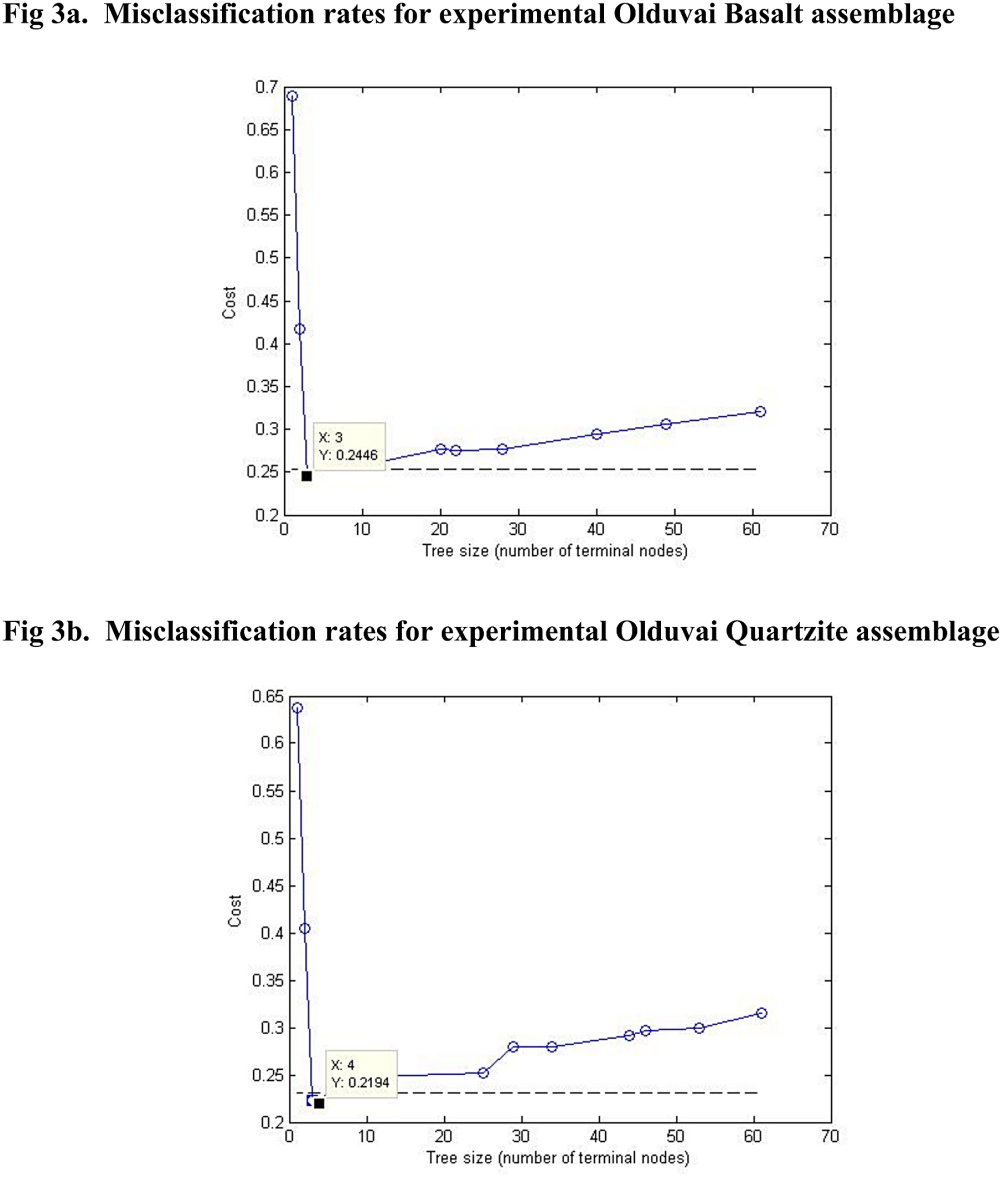
Trimming the classification tree. Each misclassification graph is constructed via random substitution of samples with empirically known classes into the trained algorithm. The X value represents the number of terminal nodes (ends of branches). The smallest value of X for the smallest misclassification value of Y is considered the most economical tree. The classification tree constructed in Fig 4 represents the most economic trees for both raw materials.

In order to differentiate experimental flakes into their constituent units, a classification algorithm was calculated using a tree function (MATLAB 2012a). Importantly, tree functions can be quantitatively used to calculate the probability of an individual classification (in this case, a flake) as belonging to a particular group (in this case, a production behavior). Misclassification rates can also be calculated for a given population in a tree function. For a given sample size, a subset of flakes was used to “train,” or construct, the classification tree function. Similar to discriminant functions, resulting trees demonstrate what metric traits best differentiate the group, in this case hierarchically. A full tree will successfully classify all flakes but is specifically trained to the data used to construct that tree. In order to use the algorithm to classify flakes of unknown behavioral origin, the tree was “trimmed” to the shortest length that statistically led to the highest level of classified flakes (Fig 3). This set of flakes was then used to build a classification algorithm for future flakes with unknown behavioral affiliation. A subset of random flakes withheld from the original sample was then tested against the constructed algorithm to calculate the misclassification rate for the classification algorithm.

**Fig 4:**
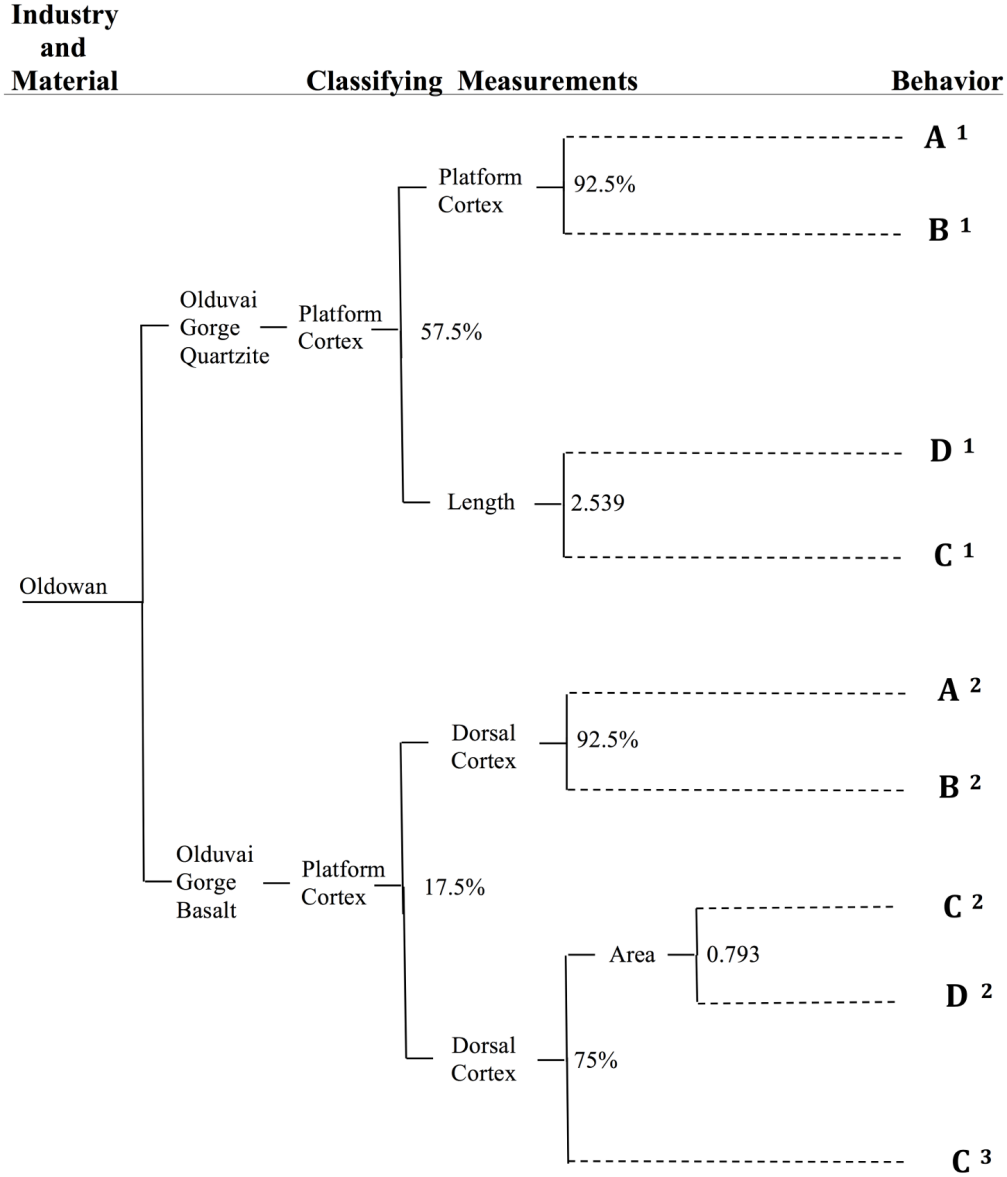
Behavioral classification for Oldowan artifacts produced on Olduvai Gorge basalt and quartzite raw materials. At each junction of the classification tree, if a given measurement is equal to or larger than the listed value, then the classification moves up the tree; if a given measurement is less than the listed value at the junction, then the classification moves down the tree until an ultimate behavioral classification is reached. For Olduvai Gorge quartzite and basalt, dorsal and platform cortex account for a large amount of the variation leading to classification. This relationship may or may not continue for other raw materials utilized at different Oldowan sites and is expected to vary in other technologies.

The experimental research presented here produced a sample of 1758 flakes from 219 cores. The classification tree based on this least-effort experimental model is presented in Fig 4. A size-standardized value for one variable is associated with each node on the tree. To classify a given flake, if the measure of that flake is equal to or larger than the value at the node, classification proceeds up the tree; if the measure of that flake is smaller than the value at the node, classification proceeds down the tree. Each behavioral classification has a superscripted number associated with it. This number refers to the misclassification rate associated with that particular behavioral classification and can be cross-referenced with the misclassification table in Table 2. Thus each flake has a dynamic production behavior associated with it and a quantified expectation for parameters of certainty for that behavioral classification

**Table 2:** Probability of correct classification for individual flakes (see Fig 4).

Classified Behavior	Raw Material	Probability A (%)	Probability B (%)	Probability C (%)	Probability D (%)
A^1^	Olduvai Quartzite	**84.00**	11.50	3.21	1.28
A^2^	Olduvai Basalt	**87.60**	7.75	1.55	3.10
B^1^	Olduvai Quartzite	8.50	**87.60**	1.77	1.77
B^2^	Olduvai Basalt	5.11	**90.30**	2.27	2.27
C^1^	Olduvai Quartzite	0.86	3.88	**65.90**	29.30
C^2^	Olduvai Basalt	0.00	0.00	**85.70**	14.30
C^3^	Olduvai Basalt	0.00	3.50	**74.50**	22.00
D^1^	Olduvai Basalt	16.70	0.00	0.00	**83.30**
D^2^	Olduvai Basalt	0.80	0.80	41.50	**56.90**

The large sample size of experimental flakes also establishes expectations for the average number of flakes a given raw material type can be expected to yield if a core is utilized to exhaustion (Table 3). This measure provides a baseline expectation for the number of expected cores and flakes at a given archaeological site if least effort methods were utilized archaeologically.

**Table 3:** Average number of flakes removed per core and average number of production behaviors per core for each raw material.

Raw Material	Average # Flakes Per Core	OBA	OBB	OBC	OBD
Olduvai Basalt	8.67	1.33	1.90	2.02	1.23
Olduvai Quartzite	9.60	1.54	2.25	1.62	0.79

## Results

**Fig 5:**
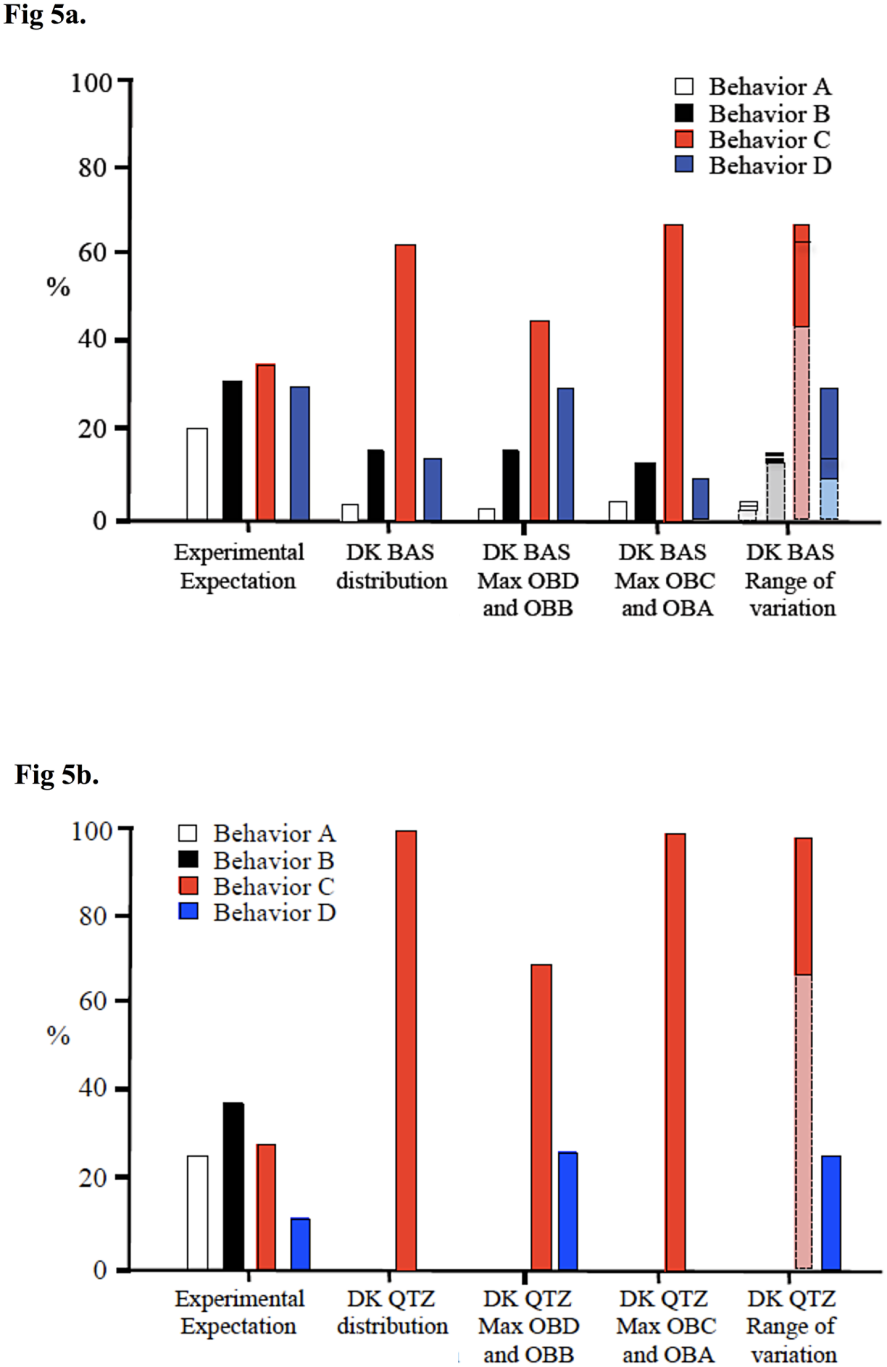
Experimental vs. archaeological distribution of Oldowan stone tool manufacturing behaviors and associated classification error margins. The first distribution (“Experimental Expectation”) represents the distribution of behaviors empirically determined from the controlled experimental replications. If all of the flakes produced at a site were produced in a least effort manner and were still present at the site when it was excavated, the archaeological distribution is expected to be similar to that of the experimental distribution. The second distribution is archaeological and classified via the classification algorithm Fig 4. However, each flake classified via this algorithm has a misclassification statistic associated with it (Table 2). Therefore, the third distribution (“Max OBD and OBB”) and the fourth distribution (“Max OBC and OBA”) demonstrate the potential extremes of the archaeological distribution given these misclassification rates. Finally, the last distribution (“Range of Variation”) combines the second, third, and fourth distributions into one combined statistic. For each bar on the graph, the dark colors represent the potential range of representation for that production behavior, given the potential misclassifications outlined in Table 2. The line inside the colored box represents the actual archaeological value, given classification via Fig 4. If no line is present in the colored box, then either the upper or lower extreme is the actual classified value. (Chi-square results: Expected vs. DK: Yates’ chi-square = 8.58, df = 1, Yates’ p = <0.01).

The first step in applying the experimental model to archaeological lithic artifacts is to statistically establish the relationship between the reduction method used in the experimental setting and the morphologies of archaeological materials. As was previously discussed, the experimental replication methods implemented here utilize the least effort approach to flake manufacture to produce a null model of Oldowan stone tool production. The null hypothesis that is tested here is that there is no statistical difference between flakes replicated using a least effort approach to production and archaeological flakes (Fig 5). If the null hypothesis is validated, then the least effort approach can be considered a valid explanation for Oldowan stone tool production. If the null hypothesis is invalidated, at least a portion of the archaeological material was produced using a reductive approach other than the least effort approach. An invalidated null hypothesis necessitates further statistical examination to define what archaeological artifacts fit within expectations for a least effort approach and what artifacts do not fit within that expected variation. For those artifacts that fit within expected variation, as established via the reduction experiments, the experimentally derived classification algorithm is used to assess what behaviors are represented. Table 4 demonstrates that while there is significant overlap between experimental and archaeological morphology, a subset of archaeological flakes from DK, both basalt and quartzite, deviate from the expectation.

**Table 4:** Distribution of assemblage variation for each measurement and raw material for experimentally produced flakes. (SL: Size-Standardized Length; SW: Size-Standardized Width; ST: Size- Standardized Thickness; SMAX: Size-Standardized Maximum Dimension; SBT: Size-Standardized Bulbar Thickness; SAREA: Size-Standardized Area).

Context	Material	Measurement	2 SD Below	Mean	2 SD Above	SD
Experiment	Basalt	SL	0.802	1.672	2.542	0.435
Experiment	Quartzite	SL	0.864	1.689	2.513	0.412
Experiment	Basalt	SW	1.084	1.940	2.796	0.428
Experiment	Quartzite	SW	0.755	1.456	2.157	0.350
Experiment	Basalt	ST	0.244	0.482	0.721	0.119
Experiment	Quartzite	ST	0.138	0.441	0.744	0.152
Experiment	Basalt	SMAX	1.628	2.489	3.349	0.430
Experiment	Quartzite	SMAX	1.599	2.352	3.106	0.377
Experiment	Basalt	SBT	0.156	0.451	0.747	0.148
Experiment	Quartzite	SBT	0.233	0.493	0.753	0.130
Experiment	Basalt	SAREA	0.355	0.563	0.771	0.104
Experiment	Quartzite	SAREA	0.399	0.677	0.955	0.139
Archaeological	Basalt	SL	0.974	1.849	2.723	0.437
Archaeological	Quartzite	SL	1.045	1.851	2.657	0.403
Archaeological	Basalt	SW	1.030	1.744	2.458	0.357
Archaeological	Quartzite	SW	1.044	1.704	2.365	0.330
Archaeological	Basalt	ST	0.326	0.570	0.815	0.122
Archaeological	Quartzite	ST	0.301	0.561	0.821	0.130
Archaeological	Basalt	SMAX	1.571	2.385	3.198	0.407
Archaeological	Quartzite	SMAX	1.601	2.352	3.103	0.375
Archaeological	Basalt	SBT	0.215	0.476	0.737	0.130
Archaeological	Quartzite	SBT	0.226	0.482	0.738	0.128
Archaeological	Basalt	SAREA	0.305	0.557	0.809	0.126
Archaeological	Quartzite	SAREA	0.344	0.572	0.800	0.114

### DK Basalt

Based on experimentally established expectations of Olduvai Gorge basalt assemblages, it is expected that given a least effort approach production strategy, flake assemblages will be slightly dominated by OBC flakes, followed by nearly equivalent numbers of OBB and OBD flakes, while OBA flakes will be the least numerous (Fig 5, “experimental expectation”). Fig 5 also provides a graphical representation of the expected range of behavioral classification given the misclassification rates calculated in the construction of the classification algorithm (Fig 4 and Table 2). While in every misclassification scenario for DK the proportion of OBC basalt flakes is higher than expected, the relative proportions of behaviors falls within expected variation (OBC is highest, followed by nearly equivalent numbers of OBB and OBD, and the lowest numbers of OBA).

The archaeological core assemblage from DK contains a total of 106 cores, 97 produced on basalt [2]. The total *debitage* from DK (this is liberally calculated as being inclusive of whole flakes and broken flakes, but exclusive of core fragments) includes 506 basalt flakes. Based on the experimental data in Table 5, if every basalt core was reduced using a least effort approach strategy and was reduced until the core was relatively exhausted of usable platforms, it would be expected that approximately 841 basalt flakes would have been produced (97 cores x 8.67 flakes/core = 841 flakes). There is a discrepancy, therefore, between expected flake counts and the archaeological flake counts. Only 60.2% of the expected number of basalt flakes was recovered.

**Table 5:** Archaeological samples of cores per site compared to the experimentally determined expectations of flakes per core (see Table 4) and the actual number of archaeological flakes recovered from the site.

Site	Raw Material	Cores (N)	Expected Number of Flakes	Actual Number of Flakes	Difference (%) (Actual/Expectedx100)
DK	Basalt	97	841	506	60.2
DK	Quartzite	9	86	233	270

The site where flakes were being produced, hominins could have carried cores that were partially reduced elsewhere into the site, in which case it is not expected that the expected number of flakes would be higher than the actual number of flakes produced on site. Third, hominins could have stopped flake production prior to a core’s exhaustion or removed flakes inefficiently.

If hominins were selectively transporting basalt flakes away from DK and to another location, it would be assumed that another contemporaneous archaeological site would show more basalt flakes than expected given the number of cores present. The location of DK (adjacent to a riverbed and farther toward the eastern volcanic highlands than other Oldowan sites at Olduvai Gorge) is such that basalt cobbles were available for hominin use in closer proximity to the site as compared to other Olduvai Gorge sites (see [54]). This relative ease of raw material procurement at DK suggests that the second scenario, that cores were reduced elsewhere and then brought into the site, is not likely.

This argument informs the third scenario as well. Economic expectations for raw material use suggest that individuals will not give particular value to a raw material that is easy to obtain; as raw materials become more difficult to obtain, more economically advantageous usage of that material is expected to increase [14,52,62,63]. Therefore, the explanation that Oldowan producing hominins stopped core reduction prior to exhaustion is the most likely explanation for the discrepancy between the expected number of basalt flakes, given the number of cores present, and the actual number of flakes present at the site. Said another way, there was not an economic reason for Oldowan producing hominins at DK to reduce basalt cobbles to the point of exhaustion. Instead, because basalt was readily available, hominins likely discarded cobbles while they still had available platforms, especially if the quality of a cobble was less than desirable. Rather than the expected average of 8.67 flakes per basalt core, it appears that at DK, hominins were removing an average of 5.2 flakes per core.

**Fig 6:**
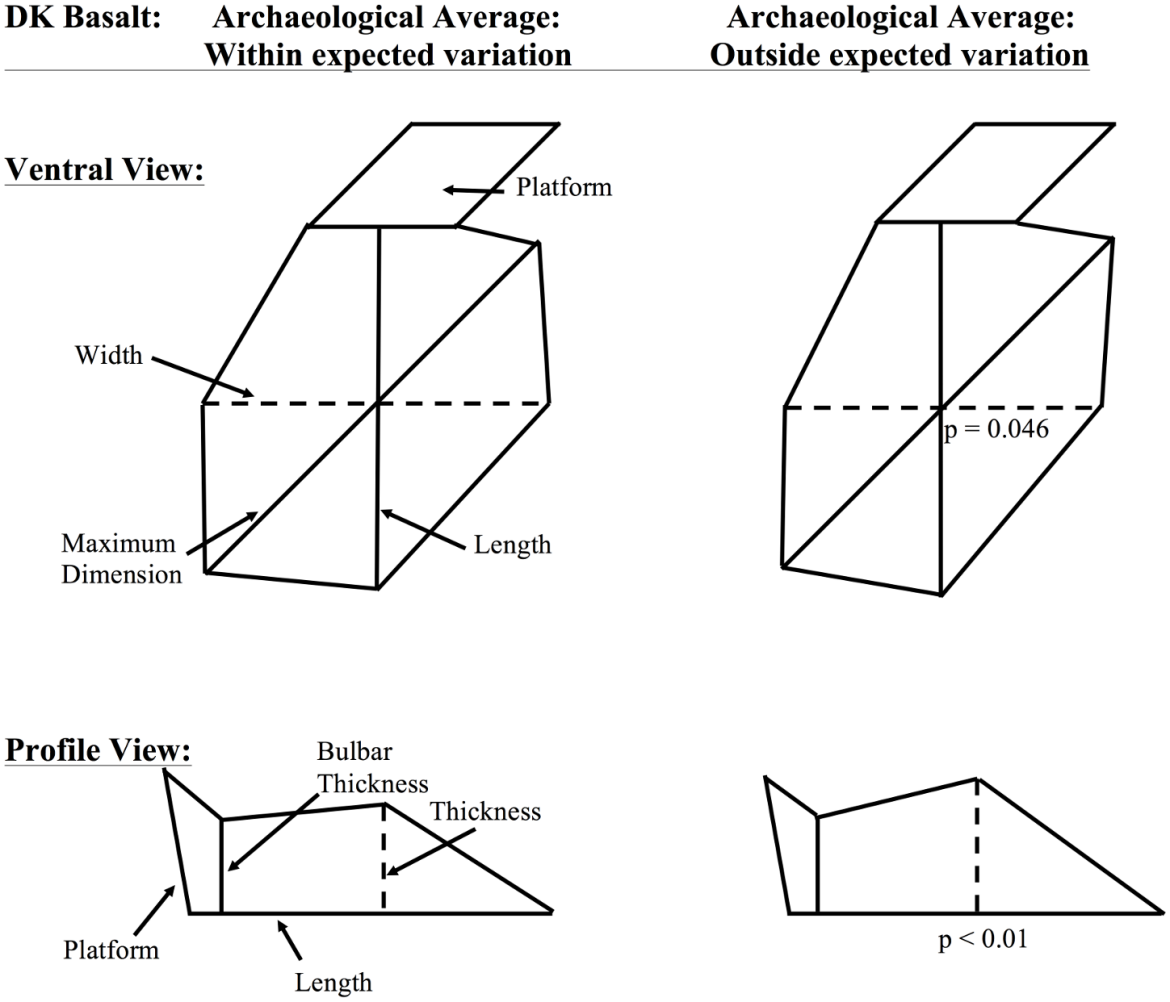
Schematic images of average basalt flakes from DK, Olduvai Gorge falling within and outside of expected variation. These images represent idealized, schematic images of average measurements for Olduvai Gorge archaeological flakes from the site of DK that are made from basalt. The first column shows the ventral and profile views of the average DK flakes that fall within experimentally expected variation. The second column shows the ventral and profile views of the average DK flakes that fall outside of experimentally expected variation. For consistency and ease of comparison, the maximum dimension was placed at a 45-degree angle from the length measurement. All measurements are still size standardized, but are translated into inch units, such that if the average size-standardized length measurement is 1.0, this becomes 1.0 inch in this image. Similarly, average platform area is depicted as a square, such that platform width and thickness are equal and both are the square root of average platform area. Significant differences between outside-of-range and within-range assemblages are dotted lines, p-values are listed.

This economic explanation for DK flake counts is reinforced by several lines of evidence: 1) deviation from flakes with morphologies suggestive of least effort approach production (Fig 6), 2) the relative proportions of production behaviors present at DK (Fig 5), and 3) the relative numbers of core types present at DK (see [2]).

For the first line of evidence, results from DK indicate that the archaeological basalt assemblage contains a subset of flakes that fall outside of experimental expectations given a least effort approach to flake manufacture. DK basalt flakes falling outside of expected variation demonstrate a clear trend of being produced in an economically inefficient manner. Fig 6 shows that these flakes significantly vary from expectations in the dimensions of width and thickness. In both cases, flakes falling outside of least-effort expectations are made thicker and less wide than expectations predict. In other words, these flakes show a decrease in perimeter and an increase in raw material used to produce the flake, which is an economically inefficient use of raw material.

By revisiting Fig 5, the second line of evidence is apparent in that all of the misclassification scenarios have a higher-than-expected percentage of OBC flakes in the assemblage. As was explained in the methodology section of this paper, a series of OBC flakes leads to a core form that M.D. Leakey would call a “bifacial chopper.” Flaking bifacially allows for flakes to be produced with relatively less energy and more precision given the non-cortical platforms utilized for their production. However, experimental data presented here suggest that OBD flakes tend to be more invasive than OBC flakes and thus more regularly produce new platforms. OBC flakes tend to limit the total number of flakes that can be removed from a core due to the fact that a core becomes thicker as bifacial flakes approach the center of that core.

Third, the quartz examples of cores at DK show more substantial flaking than basalt flakes and prompted M.D. Leakey to comment that these quartz cores “…are remarkable for the refinement of workmanship” [2]. Core types like “side choppers,” “end choppers,” and “two-edged choppers” in which flaking is minimal, are produced entirely on basalts at DK, while quartz core forms tend to be more heavily reduced.

### DK Quartzite

Based on experimentally established expectations of Olduvai Gorge quartzite assemblages, it is expected that given a least effort approach production strategy, flake assemblages will be slightly dominated by OBB flakes, followed by nearly equal numbers of OBC and OBA flakes, while OBD flakes will be the least numerous (Fig 5b, “experimental expectation”). The reason for the experimental difference between Olduvai basalt behavioral expectations (Fig 5a) and Olduvai quartzite behavioral expectations is primarily due to the fracture qualities and relative core shapes of the two raw materials. While basalt is found mainly as angular and river-rounded cobbles, the Naibor Soit quartzite outcrops in a tabular formation and breaks, primarily, into blocky pieces with square edges that were utilized for flake production. The result is that flakes tend to be invasive for the basalt cobbles, which leads to the natural formation of new platforms. For quartzite, however, the squared edges and crystalline nature of the material leads to square-edged and side-struck flakes that do not leave an invasive flake scar. Leakey [2] notes the fact that at DK, “…*all* the quartz flakes…are divergent; that is, splayed outwards from the striking platform” (emphasis added). In other words, all quartzite flakes show side-struck features due to the lack of available natural or produced areas of high mass to follow during flake manufacture. Due to this lack of invasive flaking, the flake scar lacks an appropriate platform angle and is thus difficult to use as a new platform (for OBC bifacial flaking). This results in a longer series of OBA and OBB flakes.

Fig 5b demonstrates a dramatic difference from the experimental expectation. In all scenarios of misclassification, OBC flakes dominate the assemblage (and likely represent 100% of the recovered flakes). This represents a significant difference in how quartzite is being treated at DK when compared with basalt. Since these archaeological quartzite OBC flakes morphologically fall within least effort experimental expectations, they cannot be ruled out as being produced via least effort manufacture strategies. However, the lack of any other production behaviors is highly unusual and demonstrates a notable difference in the way that Oldowan producing hominins at DK were treating quartzite as compared to basalt.

Several explanations could account for why the DK quartzite assemblage is dominated by OBC flakes. First, hominins could have preferred OBC flakes and selectively transported them into the site. Second, hominins could have initially reduced quartzite cobbles elsewhere, leaving OBA and OBB flakes behind, and brought in cores that were prepared to be flaked in a bifacial manner. Third, OBA and OBB flakes could have selectively been transported away from the site.

To establish the likelihood of each of these scenarios, it is pertinent to address the number of quartzite cores and flakes actually found at DK. From M.D. Leakey’s monograph [2], DK only has nine excavated quartz cores. Based on experimental expectations of exhausted cores (Table 5), it is expected that nine quartz cores would yield a total of 86.4 flakes. However, a total of 233 quartzite flakes and broken flakes were recovered from DK. This represents an increase of 270% from the experimentally established expectations of the number of flakes per core. This is a striking departure from the pattern formed with the DK basalt, in which only 60.2% of the expected basalt flakes are present. An overabundance of quartzite flakes suggests that 1) flakes could have been brought into the site from elsewhere (but not the cores that those flakes were produced from), 2) quartzite cores could have been removed from DK, while the flakes were left behind, or 3) quartzite cores were reduced in a highly efficient manner and only produced OBC flakes. If quartzite cores were reduced on-site at DK, it is unlikely that only OBC flakes would be produced, unless cores were specifically shaped elsewhere, which is an unlikely hypothesis for Oldowan stone tool production and one that certainly deviates from least effort expectations. Similarly, it is unlikely that all OBA, OBB, and OBD flakes would be removed from the site. This combined evidence suggests that quartzite cores were not being reduced, or were only minimally reduced, at DK. Rather, OBC flakes were being transported into the site after being produced elsewhere.

Quartzite outcrops at the Naibor Soit inselberg at Olduvai Gorge (Fig 1), a static resource that would require physical transport from Naibor Soit to the sites in question. When an exponential decay model is applied to Olduvai Oldowan quartzite artifacts [14], as a general trend, quartzite artifacts become less common and smaller as the distance increases from the raw material source. However, they also find that this trend is mediated by ecological factors surrounding site locations (as predicted by [9]). Quartzite can, therefore, be considered a costly material for stone tool producing hominins at Olduvai Gorge. It is predicted based on raw material economics that costly materials will be utilized more efficiently as the distance to the source location increases. Oldowan producing hominins may have preferred quartzite for tasks requiring a durable cutting edge [10], but acquiring quartzite was costly for hominin populations. Such functional explanations for quartzite utilization will require further experimental evidence, but research suggests that quartzite may have had a functional advantage in terms of its sharpness and durability [10]. However, quartzite shatters easily, which would be potentially dangerous if flakes were utilized to procure meat resources for consumption.

**Fig 7:**
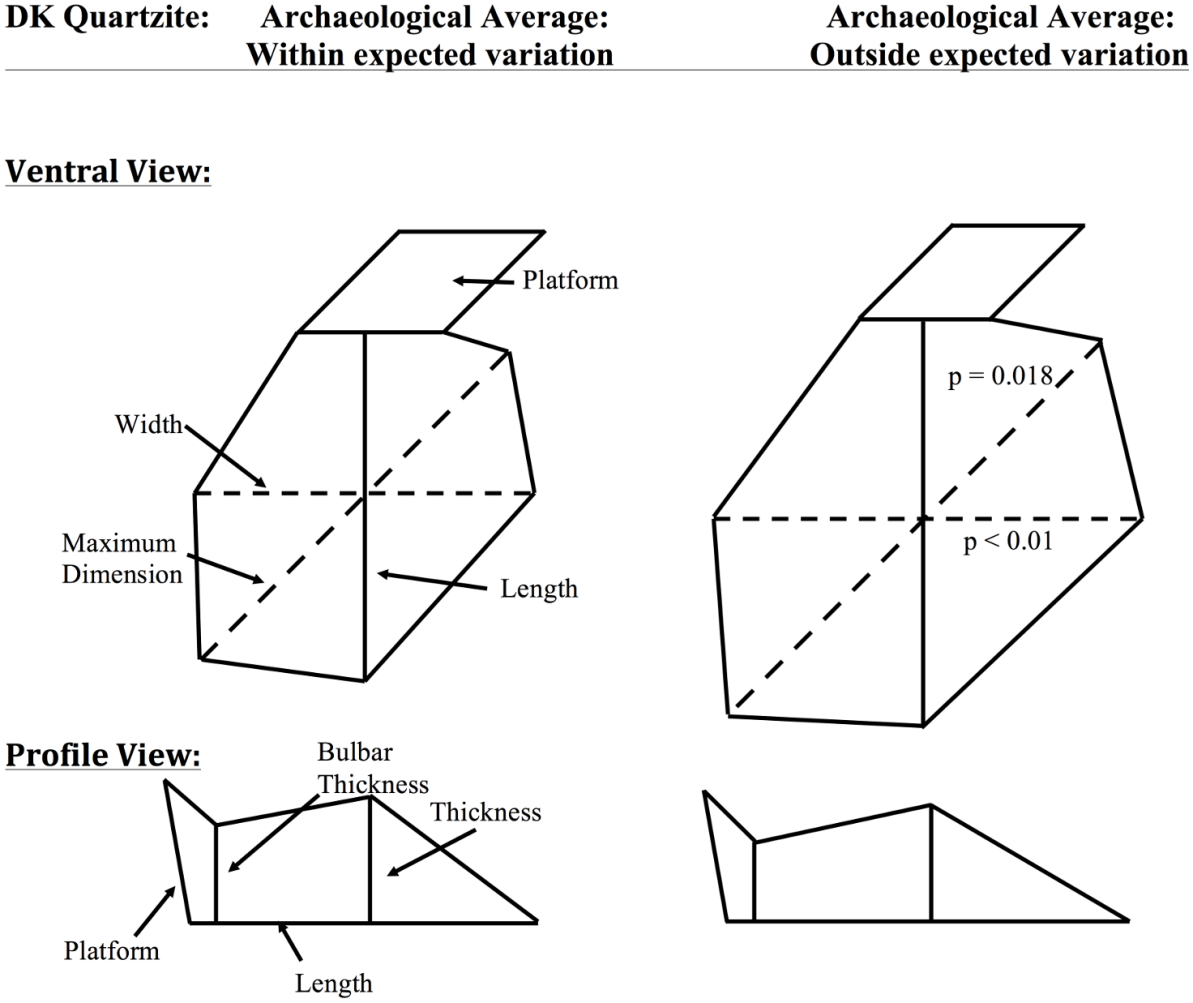
Schematic images of average quartzite flakes from DK, Olduvai Gorge falling within and outside of expected variation. These images represent idealized, schematic drawings of average measurements for Olduvai Gorge archaeological flakes from the site of DK that are made from quartzite. The first column shows the ventral and profile views of the average DK flakes that fall within experimentally expected variation. The second column shows the ventral and profile views of the average DK flakes that fall outside of experimentally expected variation. For consistency and ease of comparison, the maximum dimension was placed at a 45-degree angle from the length measurement. All measurements are still size standardized, but are translated into “inch” units, such that if the average size-standardized length measurement is 1.0, this becomes 1.0 inch in this image. Similarly, average platform area is depicted as a square, such that platform width and thickness are equal and both are the square root of average platform area. Significant differences between outside-of-range and within-range assemblages are dotted lines, p-values are listed.

Results from Fig 7 corroborate this economic interpretation. While previous research demonstrates that the *absolute* maximum dimension of quartzite artifacts decreases as distance to Naibor Soit increases [14], results presented here (Fig 7) demonstrate that *relative* maximum dimension and width of flakes actually increases at DK. For quartzite flakes that fall outside of expected least effort variation, hominins were able to consistently create flakes that had a relatively larger perimeter (increased maximum dimension and width) while preserving the same platform dimensions and relative thickness. This demonstrates an economically efficient use of raw material and directly opposes the way that basalt was being treated at the same site.

Most importantly, this deviation from expectation shows a continuous ability of Olduvai hominins to produce flakes in a manner that cannot be considered a least effort approach to stone tool production. For quartzite, this ability demonstrates an economic understanding of quartzite utility since hominins are creating flakes in a more efficient manner than least effort predictions. This contrasts significantly with how Olduvai hominins were interacting with basaltic materials. For basalt, hominins were also producing some flakes in a way that deviates from least effort expectations. However, these flakes were produced in an economically inefficient manner, suggesting that hominins did not economically value the material. The placement of DK at the far eastern side of Olduvai Gorge and its landscape location adjacent to a paleoriver suggest that cobbles were not far from the site and thus demonstrates a differential value placed on raw materials during the Oldowan at DK.

## Discussion

Experimental models in archaeology have proven an effective research method for a broad array of archaeological scenarios through time and space [3,4,7,8,10,13,28,34,35,39,40,41–47,56,64–74]. Of course, such models necessarily make assumptions [75–78], but the trade for these assumptions is a statistical foundation, a set of empirically determined expectations, and ideal conditions. Most importantly, models provide the parameters by which null hypotheses can be tested. The experimental model presented here establishes an expectation for the lithic production behaviors of Oldowan hominins at Olduvai Gorge on basalt and quartzite raw materials. By producing assemblages of flakes on indigenous Olduvai Gorge raw materials, each with empirically known and recorded sets of behaviors, a large and complete assemblage of Oldowan flakes was created. Without some such empirical model for what morphological range might be expected within the Oldowan, assemblage comparison of variation is not grounded on any quantitative measure. The model established via the methodology outlined here removes qualitative conclusions and replaces them with statistically effective measures for lithic production behaviors based on a null model.

Previous research suggests that the Oldowan is not as homogeneous as was previously assumed. The most substantial of these papers concern the stone tool production skills of hominins at Lokalalei, West Turkana [29] and of the technological variability in the Oldowan based on the early stone tools recovered from Gona, Ethiopia [33]. These studies are impactful in their scope and sample, but would benefit from an assessment of what the Oldowan is expected to look like before stating that a particular assemblage varies from such an expectation. This is not to say that the conclusions from Lokalalei or Gona are incorrect. Rather, these conclusions must be corroborated by placing them within a broader comparative framework of Oldowan technological ability. For instance, assessing whether the Lokalalei artifacts vary from least effort assumptions given the raw material that they were produced on would make these assemblage comparable to other assemblages.

Recent experimental research suggests that teaching, and potentially language, may have developed through Oldowan production skills [79]. This research provides archaeological support for such experimental hypotheses because it allows Oldowan stone tools to be investigated in terms of a known expectation for morphological variation.

For the site of DK at Olduvai Gorge, it is apparent that there is both deviation from least effort assumptions and deviation from least effort assemblage composition. For flakes falling outside of least effort experimental expectations (Fig 5), it was shown that basalt flakes were produced in a less-efficient manner than predicted and that quartzite flakes were produced in a more efficient manner than predicted (Figs 5 and 6, respectively). This differential utilization of raw materials at DK demonstrates that hominins were economically aware of their surroundings and were able to mediate procurement costs with differential patterns of flake production. Moreover, the fact that the ratio of production behaviors for quartzite flakes at DK deviates from the experimental expectation (Fig 5b) indicates that quartzite flake production did not occur onsite at DK. Rather, the dominance of quartzite OBC flakes at DK suggests that these flakes were produced elsewhere and selectively transported into the site. Economically, this is expected. Instead of Oldowan producing hominins transporting large, heavy cobbles of quartzite across the dangerous paleolandscape, these hominins likely produced quartzite flakes at the raw material source and selectively transported the resulting, light-weight quartzite OBC flakes across the landscape. It is hypothesized that quartzite OBC flakes may have had a functional advantage over OBA and OBB flakes due to their relative lack of cortex. Quartzite cortex makes flake edges more brittle and prone to breakage, though overall research has demonstrated that quartzite is a durable and functional material, especially for butchery activities [10]. Economic utilization of quartzite is also surprising given widespread usage of bipolar reduction of quartzite following Middle Bed II at Olduvai Gorge [7,50].

The methodology defined by the research presented here and demonstrated through its application to the Oldowan archaeological site of DK at Olduvai Gorge, provides a tool to quantitatively assess differential production behaviors in the Oldowan and thus provides such an expectation for variability within the Oldowan. Perhaps most importantly, this quantitative tool allows individual flakes and entire sites to be directly compared with other flakes and sites in equivalent behavioral units, independent of raw material constraints. This methodology breaks from the archaeological traditional of using typologies and metric assemblage characteristics to assess similarities and differences between sites. Instead, this methodology assumes the null hypothesis that all stone tools were made using the same method and then identifies specific flakes and assemblage-level patterns that deviate from or confirm the null hypothesis.

In developing the methods presented here, it is the hope of the author that these methods will be applied to assemblages more broadly so that vastly different assemblages might be brought into direct comparison in terms of production behaviors. Such assemblage comparison can be used to explain how technology develops at one locality through time: how do production behaviors vary between the Oldowan and Developed Oldowan at Olduvai Gorge? Or they can be used to assess how technology varies across space: how do “Oldowan” assemblages from different parts of the world deviate from each other in terms of production behaviors (for example, compared to [80–84])? Do Oldowan production behaviors statistically precede those utilized in the Acheulian at Olduvai Gorge, or does *Homo erectus* begin an entirely new process of stone tool production? Such analyses would provide a sister-strategy to addressing cladistic change in core shape [85], for instance, by focusing on flake characteristics and core reduction in addition to the shape of the core. Increasing the sample of size of experimental flakes and widening the number of experimental participants will only strengthen the model presented here. Application of these methods to a broader array of raw material types and identification of production behaviors for other Oldowan assemblages, Developed Oldowan assemblages, and early Acheulean assemblages will allow for quantitative comparison of production behaviors between sites and hominin groups. The Oldowan provides a platform from which to establish evidence for how hominin populations initially produced stone tools. From this foundation further analysis can quantitatively demonstrate how production strategies deviate from this initial strategy and begin to explain what adaptive strategies these deviations were fulfilling; in other words, the evolutionary history of stone tool production can be expressed objectively and quantitatively.

## Supporting Information

S1 File This supporting information file includes detailed calculation methods for platform area and an explanation for how experimental core morphology affects resulting flake morphology. (DOCX)
