# Supplementary material for: Quantifying Oldowan Stone Tool Production at Olduvai Gorge, Tanzania
Source: PLoS One. 2016 Jan 25;11(1):e0147352. doi: 10.1371/journal.pone.0147352 (PMC4726611; doi:10.1371/journal.pone.0147352)
Supplement: S1 File — (DOCX) [file pone.0147352.s001.docx]

**Supplemental Material**

**Calculating platform area**

The six platform variables defined in Table 1 are combined to estimate platform area. Platform area has traditionally been calculated as platform width multiplied by platform thickness [1]. This simple calculation, however, assumes a rectangular platform shape and thus may overestimate or underestimate true platform area [2].

For comparison of archaeological assemblages using the variable defined above, multivariate analyses (like Multivariate Analysis Of Variation (MANOVA)) require that variables be independent. To determine if measurements covary, covariance coefficients were calculated between each of the eleven variables. Not surprisingly given their similarity, platform measurements are not statistically independent (Table A). A measure of platform area produces a composite of these measured platform variables that can be used for statistical purposes with the other, previously described measurements. The methodology presented here requires only the use of digital calipers and thus is a mobile means of calculating platform area. This is particularly useful for measuring platform area on a large assemblage of flakes and is a reliable, quick alternative to photographic analysis of platform area.

**Table A. Covariation coefficients for platform measurements taken on all whole**

**flakes**

| **Variable** | **PW** | **LPW** | **RPW** | **PT** | **LPT** | **RPT** |
| --- | --- | --- | --- | --- | --- | --- |
| **PW** | 1.0 |  |  |  |  |  |
| **LPW** | 0.89 | 1.0 |  |  |  |  |
| **RPW** | 0.89 | 0.62 | 1.0 |  |  |  |
| **PT** | 0.68 | 0.60 | 0.81 | 1.0 |  |  |
| **LPT** | 0.64 | 0.51 | 0.62 | 0.81 | 1.0 |  |
| **RPT** | 0.61 | 0.57 | 0.53 | 0.83 | 0.69 | 1.0 |

Platform area is calculated using simple geometry. The platform measurements taken for each flake are depicted in Fig A1 and A3. The triangle formed (Triangle A) from the left platform thickness measurement (the base of the triangle) and *h* (the height of the triangle, which is 1/5 the total platform width) is calculated using the basic formula for calculating triangle area (*E1*) in which *b* is the base of the triangle and *h* is the height of the triangle:

(E1) *A = 0.5bh*

The triangle formed (Triangle B) on the right lateral side of the platform is similarly calculated using right platform thickness (*b*) and *h* (see Fig A1).

**Fig A.** a) Each platform is divided into four triangles. Triangles A and B show the variables for base (*b*) and height (*h*), used in calculating their areas. b) Simply calculating the areas of Triangles C and D leaves a large portion of the platform area out and thus underestimates total platform area. c) In order to determine the actual areas of Triangles C and D, their respective heights must first be determined. PW, LPW, and RPW are used in Heron’s Law to calculate the area of the triangle depicted here. This area is then used to calculate the height of that triangle, which is also the height of Triangle C. Subtracting BPT from PT will provide the height of Triangle D (UPT).

To approximate the remaining area between triangles A and B, the areas of triangles C and D are calculated. However, as Fig A2 demonstrates, simply calculating the area of triangles C and D underestimates the total platform area. Thus the basic triangular area equation, *E1*, is an insufficient approximation for triangles C and D. To better estimate the actual areas of the platform above and below the platform width dimension and between the left and right platform thicknesses, equation *E2* is used.

(E2) *A = xbh*

In *E2*, the base (*b*) of triangles C and D is Middle Platform Thickness (MPW), which is simply 3/5 of the total platform width. The height (*h*) of triangles C and D are Bottom Platform Thickness (BPT) and Upper Platform Thickness (UPT), respectfully. The coefficient (*x*) corrects for the underestimation of area by *E1*. To determine BPT, UPT, and *x*, the following steps are taken.

To calculate the height of triangle C (BPT), Heron’s Law is used (Fig A3). In Heron’s Law, if all three sides of a triangle are known, the following formula (*E3*) calculates the area (*A*) of the triangle, where *s* is the semi-perimeter of the triangle.

(E3)

The area of this larger triangle (seen in Fig A3) is not itself useful for calculating total platform area. This is because the lateral portions of this triangle include parts of

triangles A and B, which have already been calculated. Including this larger triangle would therefore overestimate the total area of the platform. Instead of using the area of this triangle to calculate total platform area, it is used to calculate the triangle height (BPT) of triangle C by solving *E1* for *h* (see *E4*). Upper Platform Thickness (UPT), which is the height of triangle D, is calculated by subtracting BPT from PT.

(E4)

To calculate the coefficient, *x*, three idealized versions of platforms are used to establish expected coefficients given the relative measurements of PT, LPT, and RPT (Fig B). Based on these expectations, a linear equation is derived to model any actual measurements on archaeological flakes.

**Fig B.** The figures above demonstrate platform area calculations for three idealized scenarios. a) The platform thickness, left platform thickness, and right platform thickness are all equal. In this case the coefficient used to calculate the area of Triangles C and D would be 1.0. b) The platform thickness is exactly half of the left and right platform thicknesses. In this case the coefficient used to calculate the area of Triangles C and D would be 1.5. c) The platform thickness is exactly twice the size of left and right platform thickness. In this case the coefficient used to calculate the area of Triangles C and D would be 0.75. Of course, platform dimensions rarely fit such parameters. The theoretical scenarios presented here (and in arbitrary units in Table B) provide a means to construct a general equation to model any platform based on the relative platform thicknesses.

The first idealized version is a scenario in which PT = LPT = RPT. In this scenario, using equation *E1* would omit the light gray platform areas. To more accurately estimate the areas of triangles C and D, the formula below is used:

(E5) *A = 1.0bh*

This is simply the product of (length x width), which is the total area of the rectangles depicted in Fig B1. In this case, the coefficient (*x*) used for the area equation is 1.0.

The next idealized scenario (Fig B2) is when PT = 0.5LPT =0.5RPT. In this scenario, using formula *E6* is appropriate and is a close approximation of platform area when added to the areas of triangles A and B. In this case, the coefficient (*x*) used for the area equation is 1.5.

(E6) *A = 1.5bh*

The final idealized scenario (Fig B3) is when PT = 2LPT = 2RPT. In this case, the formula calculating area of a rectangle (A=BH) overestimates the upper

and lower areas of the platform, but the formula calculating area of a triangle (*E1*) underestimates the upper and lower areas of the platform. However, using a coefficient (*x*) of 0.75 (*E6*) approximates platform area well, as seen in Fig C3.

(E7) *A = 0.75bh*

Of course, actual flake platforms will have any combination of relative platform thicknesses. Using the idealized scenarios described above, the average of the LPT and the RPT was calculated, and then the ratio between PT and this average was determined for each scenario using an arbitrary, unitless value of “10” as the standard (Table B). Each of these idealized scenarios has a known coefficient used to calculate area. The ratio between PT and the average Left and Right Platform Thickness is plotted against the idealized coefficient in Fig C. This linear relationship can be described through the linear formula (*E8*):

(E8) *y = 0.5x + 0.5*

**

**Fig C.** The points plotted here demonstrate the relationship between variable platform thicknesses and the coefficient that best estimates area given that relationship. The plotted points are derived from the idealized scenarios in Fig B and the hypothetical values in the last two columns in Table B of file S1.

**Table B**. **Idealized relationship between platform thickness values and the coefficient used to calculate the areas represented by triangles C and D in Figure 3**

| **PT** | **LPT** | **RPT** | **X*** | **X/PT** | **Coefficient** |
| --- | --- | --- | --- | --- | --- |
| 10 | 10 | 10 | 10 | 1.0 | 1.0 |
| 5 | 10 | 10 | 10 | 2.0 | 1.5 |
| 20 | 10 | 10 | 10 | 0.5 | 0.75 |

* X = Average of LPT and RPT (LPT+RPT/2)

The value of *x* is the ratio between PT and Left and Right Platform thickness and is thus empirically known via measurements. The dependent variable is the coefficient, *y*, which is calculated and then used as the coefficient in the original equation, *E2,* to calculate the area of triangles C and D. For triangle C, MPW is *b* and BPT is *h*; for triangle D, MPW is *b*, and UPT is *h*.

For each flake, the coefficient for the area formula was calculated to produce area approximations for triangles C and D. These areas were added to the areas calculated for triangles A and B to get a value for Total Platform Area (PA). The accuracy of this estimation of platform area was tested using digital software. Digital photographs were taken of the platforms of a random sample of 40 replicated flakes. Platforms varied in terms of shape, size, and cortical covering. A Benchmark photographic stand ensured stable lighting and clear photographs taken with a Canon EOS T2i digital camera. A metric scale was used for each photograph and was placed at the same focal height as the platform (+/- 1.0 mm) to ensure that issues of parallax would not affect scaling [3,4]. Images were imported into the computer program ImageJ Version 1.45s [5] for analysis. Each platform was digitally outlined, thus providing an empirical assessment of actual platform area. These measures of platform area were plotted against the platform area that was calculating by using *E2* and the methods outlined above. Fig D demonstrates that actual and estimated platform areas are highly correlated (r^2^ = 0.9946). This relationship is statistically significant, demonstrating that no difference exists between the two samples (Mann-Whitney U-Test, p=0.98 for alpha = 0.01).

**Fig D.** Empirically derived values for Platform Area were determined via ImageJ

and were plotted against estimated Platform Area values determined via the derived equation, *E2*. Results show significantly correlated results (r^2^ = 0.9946).

Equation *E2*, as derived from the idealized platforms depicted in Fig B, thus provides an accurate measure of platform area. This technique provides an efficient and mobile method of calculating platform area for this and future studies.

**Core morphology and influence on flake morphology**

To assess whether the initial core morphology of the quartzite and basalt materials utilized in this study differentially influenced the resulting assemblage of flakes, cores were classified according to their shape (Fig E and Table C). A Kruskal-Wallis and Tukey’s post hoc test for multiple comparisons was conducted to determine whether cores of different initial morphological classification tend to differentially yield more or fewer total flakes. Results demonstrate no significant relationship (Table D and E).

**Table C. Metrics for core shape category determination**

| **Category** | **Length : Width** | **Thickness : Length** | **Description** |
| --- | --- | --- | --- |
| 1 | <1.5 | >0.65 | Rounded Edges |
| 2 | <1.5 | >0.65 | Blocky/Angular Edges |
| 3 | <1.5 | <0.65 | Discoidal |
| 4 | >1.5 | <0.65 | Oblong |

**Fig E.** Core morphology classification system to determine the effects that initial core morphology had on the resulting flake assemblage. Cores were measured according to the measurements in Table 1.

**Table D. Kruskal-Wallis test for multiple comparisons results testing whether cores of different initial morphological classification differentially yield more or fewer total flakes**

| **Site** | **Material** | **p** |
| --- | --- | --- |
| Olduvai Gorge | Basalt | 0.26 |
| Olduvai Gorge | Quartzite | 0.24 |

**Table E. Tukey’s post-hoc test for multiple comparisons results testing whether cores of different initial morphological classification differentially yield more or fewer total flakes. No results below are significant.**

| **Core category comparison** | **Material** | **Lower confidence interval** | **Difference in group means** | **Upper confidence interval** |
| --- | --- | --- | --- | --- |
| 1 vs. 2 | Basalt | -65.45 | -26.48 | 12.50 |
| 1 vs. 3 | Basalt | -43.36 | -17.83 | 7.70 |
| 1 vs. 4 | Basalt | -46.80 | -14.38 | 18.05 |
| 2 vs. 3 | Basalt | -23.02 | 8.64 | 40.30 |
| 2 vs. 4 | Basalt | -25.34 | 12.10 | 49.54 |
| 3 vs. 4 | Basalt | -19.67 | 3.46 | 26.59 |
| 1 vs. 2 | Quartzite | -17.69 | 25.17 | 68.03 |
| 1 vs. 3 | Quartzite | -27.13 | 11.01 | 49.14 |
| 1 vs. 4 | Quartzite | -39.28 | 3.58 | 46.44 |
| 2 vs. 3 | Quartzite | -37.31 | -14.16 | 8.99 |
| 2 vs. 4 | Quartzite | -51.89 | -21.58 | 8.72 |
| 3 vs. 4 | Quartzite | -30.57 | -7.42 | 15.72 |

**References:**

[1] Dibble HL, Pelcin A. The effect of hammer mass and velocity on flake mass. J Archaeol Sci. 1995; 22: 429-439.

[2] Shott MJ. The quantification problem in stone-tool assemblages. Am Antiquity. 2000; 65: 725-738.

[3] McPherron SP, Dibble HL. Stone tool analysis using digitized images: examples from the Lower and Middle Paleolithic. Lithic Technology. 1999; 24: 38-52.

[4] Braun DR, Harris JWK. (2003). Technological developments in the Oldowan of Koobi Fora: innovative techniques of artifact analysis. In: Moreno JM, Torcal RM, Sainz IT, editors. Oldowan: rather more than smashing stones. Barcelona: University of Barcelona Press; 2003. pp. 117-144.

[5] Rasband WS. ImageJ. U.S. National Institutes of Health, Bethesda, MD, USA. 2007. http://rsb.info.nih.gov/ij/index.html
